# Subdominant Antigens in Bacterial Vaccines: AM779 Is Subdominant in the *Anaplasma marginale* Outer Membrane Vaccine but Does Not Associate with Protective Immunity

**DOI:** 10.1371/journal.pone.0046372

**Published:** 2012-09-28

**Authors:** Saleh M. Albarrak, Wendy C. Brown, Susan M. Noh, Kathryn E. Reif, Glen A. Scoles, Joshua E. Turse, Junzo Norimine, Massaro W. Ueti, Guy H. Palmer

**Affiliations:** 1 Paul G. Allen School for Global Animal Health, Washington State University, Pullman, Washington, United States of America; 2 Department of Veterinary Microbiology and Pathology, Washington State University, Pullman, Washington, United States of America; 3 Animal Diseases Research Unit, Agricultural Research Service, United States Department of Agriculture, Pullman, Washington, United States of America; University of Minnesota, United States of America

## Abstract

Identification of specific antigens responsible for the ability of complex immunogens to induce protection is a major goal in development of bacterial vaccines. Much of the investigation has focused on highly abundant and highly immunodominant outer membrane proteins. Recently however, genomic and proteomic approaches have facilitated identification of minor components of the bacterial outer membrane that have previously been missed or ignored in immunological analyses. Immunization with *Anaplasma marginale* outer membranes or a cross-linked surface complex induces protection against bacteremia, however the components responsible for protection within these complex immunogens are unknown. Using outer membrane protein AM779 as a model, we demonstrated that this highly conserved but minor component of the *A. marginale* surface was immunologically sub-dominant in the context of the outer membrane or surface complex vaccines. Immunologic sub-dominance could be overcome by targeted vaccination with AM779 for T lymphocyte responses but not for antibody responses, suggesting that both abundance and intrinsic immunogenicity determine relative dominance. Importantly, immunization with AM779 supports that once priming is achieved by specific targeting, recall upon infectious challenge is achieved. While immunization with AM779 alone was not sufficient to induce protection, the ability of targeted immunization to prime the immune response to highly conserved but low abundance proteins supports continued investigation into the role of sub-dominant antigens, individually and collectively, in vaccine development for *A. marginale* and related bacterial pathogens.

## Introduction

Vaccines are the most effective means to control infectious diseases of humans and animals. The overwhelming majority of vaccines have been developed by one of two means: the pathogen is killed, and thus unable to establish infection, or a live attenuated strain of the specific pathogen is used to establish transient infection but without disease. While these classic approaches have been used successfully to prevent disease, there remain numerous bacterial, viral, and parasitic pathogens for which these approaches have not been successful. Identifying the specific antigens required for immunity has been an overarching goal in vaccine discovery and development over the past 30 years. Identification of specific antigens and associated mechanisms of immunity offers the promise of focusing the immune response on the key targets as well as developing lower-cost vaccines in which the specific required component is produced synthetically. There has been success: the development and use of the *Haemophilus influezae* type B vaccine, composed of a specific polysaccharide antigen and a protein conjugate, has reduced *H. influenza* meningitis in the United States by 98% and has had similar impact in other countries where childhood vaccination has become routine [1].

The availability of complete genome sequences of pathogens and the linkage of genome data to higher throughput proteomic and immunologic approaches has accelerated the identification of the full set of possible antigens involved in protective immunity [2]. We have pursued these approaches for *Anaplasma marginale*, a bacterial pathogen of wild and domestic ruminants, which causes severe livestock losses, especially in sub-tropical and tropical regions worldwide, and also serves as a model for related rickettsial diseases of humans [3],[4]. Importantly, while immunization with purified outer membranes induces significant protection against bacteremia in replicate trials, protection is both variable among vaccinates, with some animals being completely protected against infection and others poorly protected [5],[6],[7]. Consequently, we seek to identify antigens in the outer membrane immunogen associated with protection and to enhance the response to these specific antigens with the goal of providing more uniform protection.

The *A. marginale* surface is characterized by the presence of two highly abundant and closely related outer membrane proteins Major Surface Protein 2 (Msp2) and 3 (Msp3) [8]. Unsurprisingly, the predominant immune responses are generated against these two proteins [9],[10],[11]. However, both Msp2 and Msp3 are highly antigenically variable, both within an infection and between strains [12],[13],[14],[15]. Thus, while antibody to Msp2 and Msp3 antigenic variants plays a key role in how persistent infection is established and the population strain structure, these abundant surface proteins are not targets for development of a widely cross-protective vaccine and anti-Msp2/Msp3 immune responses do not associate with protective efficacy of the outer membrane vaccine [16],[17]. Using genomic and proteomic approaches, we have identified the minor components of the outer membrane protein immunogen [7],[18],[19],[20],[21],[22]. Although markedly less abundant, these minor proteins are invariant during infection and highly conserved among strains—thus representing much more attractive targets for vaccine development. Importantly, the proteomic identification within the outer membrane immunogen and the bioinformatic prediction of surface localization was confirmed for a subset of these proteins by surface-specific cross-linking [7]. The isolated cross-linked surface protein complex induced protection equal to that of the native outer membrane immunogen [7].

Among these minor components of the outer membrane immunogen, we selected AM779 based on the following criteria: i) AM779 was confirmed to be present in the surface complexes when either 1.14 nm (bis[sulfosuccinimidyl] suberate) or 1.2 nm (3,3-dithiobis[sulfosuccinimidylpropionate]) cross-linkers were used in independent experiments, increasing confidence the protein is truly surface exposed [7]; ii) AM779 is highly conserved with 99–100% identity at the amino acid level among otherwise genetically and phenotypically distinct *A. marginale* strains, suggesting that a vaccine would likely protect against multiple strains [7]; and iii) AM779 has orthologs present in related species in the genera *Anaplasma* and *Ehrlichia* (e value≤10^−20^) and *Rickettsia* (e value≤10^−5^), suggesting that outcomes would also be informative for vaccine development against additional animal and human pathogens [4]. Using AM779, we tested a series of three sequential hypotheses. The first is that AM779 is immunologically sub-dominant in vaccinates immunized with either the outer membrane immunogen or the cross-linked surface complex. The importance of sub-dominant epitopes in immune protection has been clearly established for several viral pathogens [23],[24]. The second hypothesis is that immunization with recombinant AM779 will result in significantly greater enhancement of specific B and T cell responses to AM779 as compared to those induced by the more complex immunogens. If accepted, this would indicate that it is the membrane context of minor proteins such as AM779 that results in their sub-dominance rather than an intrinsic lack of epitopes. The third is that AM779 will generate recall responses upon *A. marginale* challenge. This would support that despite lower abundance in the outer membrane, there is sufficient antigen in the native context to stimulate the anamnestic response—a requirement for sub-dominant antigens to function in vaccines. Fourth, we tested whether immune responses to AM779, induced either by immunization with AM779 alone or by immunization with outer membranes or surface complexes, correlated with protection against challenge. Herein we present the results of testing these four hypotheses and discuss the significance of the results in vaccine development for *A. marginale* and related pathogens.

## Materials and Methods

### Amplification, cloning, expression and purification of AM779

AM779 was amplified from genomic DNA of the St. Maries strain of *A. marginale* using forward (5′-GGGGACAAGTTTGTACAAAAAAGCAGGCTTATCCGAGCCTCGGGAGGAG-3′) and reverse (5′-GGGGACCACTTTGTACAAGAAAGCTGGGTACTAAAAATCAAAC-3′) primers. Amplification consisted of 30 cycles with melting temperature at 94°C for 15 s, annealing at 60°C for 30 s, and extension at 72°C for 1 min. The AM779 amplicon was size-separated by agarose gel electrophoresis and visualized following staining with SYBR Safe DNA GEL Stain (Invitrogen). The amplicon was cloned into the pDONR221 vector as an entry clone and then into the Gateway pDEST™17 expression vector (Invitrogen). After overnight growth in LB broth, plasmid DNA was purified from the transformed colonies (TOP10 *E. coli* cells) using Wizard plus Miniprep DNA purification system (Promega). Plasmid inserts were sequenced in both directions using the Big Dye kit and ABI Prism automated sequencer (Applied Biosystems). Plasmid DNA bearing correctly oriented inserts was transformed into BL21-AI *E. coli*. For expression, bacteria were grown in LB broth at 37°C and induced using 0.02% arabinose. Bacteria were harvested at 5 h post induction by centrifugation, pellets were re-suspended in lysis buffer, 6 M guanidine hydrochloride, 20 mM sodium phosphate, pH 7.8, and 500 mM sodium chloride and disrupted by sonication. The recombinant protein was purified from the bacterial lysates using HisPur cobalt resin (Invitrogen). The eluted recombinant protein was dialyzed against phosphate buffered saline (pH 7.3) and stored at −80°C after adding complete Mini protease inhibitors according to the manufacturer specifications (Roche).

### Animals and immunization

The immunization of calves with either native *A. marginale* outer membranes isolated from the St. Maries strain or cross-linked and isolated surface protein complexes of the same strain has previously been reported [7]. This prior report includes the seronegative status prior to immunization, MHC class II haplotypes of each animal (n = 5 per group), the immunization procedure, the challenge with the St. Maries strain, the infection and disease parameters, and the analysis of protection. The availability of protection data from these animals provided the opportunity to correlate AM779 immune responses with the protection induced by complex membrane immunogens containing AM779. To test whether immunization with AM779 would overcome the immunologic sub-dominance observed in context of the complex immunogens, to test recall upon *in vivo* challenge, and to assess induction of protective immunity, a second set of animals were screened by Msp5 c-ELISA to confirm seronegative status and the MHC class II haplotypes determined by PCR-restriction fragment length polymorphism analysis of the DRβ3 locus as described in detail [25],[26]. This second set of animals was immunized with either 20 µg isolated outer membranes (n = 5) in saponin, 20 µg recombinant AM779 protein in saponin (n = 5), or the adjuvant alone. The immunization procedure including antigen and adjuvant dose and route of delivery was similar to that reported for the first set [7].

### Determination of antibody titers

Antibody titers were determined for AM779 and Msp2 by immunoblotting. Briefly, either individual recombinant proteins or native *A. marginale* outer membranes isolated from the St. Maries strain as previously described [7] were separated by SDS polyacrylamide gel electrophoresis using 4–20% precast gels (BioRad). Recombinant Rap-1 (from *Babesia bovis*, a pathogen not found in the U.S.) was used as a negative antigen control. Proteins were transferred to polyvinylidene fluoride (PVDF) membranes which were blocked for 1 hr in I-Block (Applied Biosystems) blocking reagent containing 0.5% Tween 20. Individual sera from animals immunized with either outer membranes (n = 10), surface protein complexes (n = 5), AM779 (n = 5), or saponin adjuvant in the absence of antigen as a negative control (n = 5), were serially diluted and tested individually to establish endpoint titers. Binding was detected following incubation with HRP-conjugated sheep anti-bovine IgG2 antibody (Serotec) diluted 1∶20,000 by using an Amersham ECL Plus Western blotting system (GE Healthcare).

### Determination of T lymphocyte response

To evaluate T cell responses, PBMC were isolated as previously described from animals immunized with either outer membranes (n = 5) or AM779 (n = 5) [6]. Cells were assayed in triplicates wells with 1 or 3 µg per ml of antigen, either the isolated outer membrane preparation or purified recombinant AM779. As all animals had been immunized with a clostridial bacterin (Vision® 8/Somnus with Spur®, Intervet) as part of routine preventive care, the vaccine antigen was used as a positive control; recombinant *B. bovis* Msa-1 protein was used as a negative control. After six days of stimulation, cells were radiolabeled with 0.25 µCi of [^3^H] thymidine (Dupont New England Nuclear) for 12 to 18 hours. The cells were then harvested into glass filters, and the incorporation of radiolabeled nucleotides was measured with a Beta-plate 1205 liquid scintillation counter (Wallac). The stimulation index (SI) was calculated as the mean counts per minute (cpm) of cells cultured with the test antigen/mean cpm of cells cultured with the negative control antigen MSA-1.

### Infectious challenge

The second set of vaccinates was challenged by adult male *Dermacentor andersoni* infected with the St. Maries strain of *A. marginale* [27]. Ticks were infected by acquisition feeding for seven days on an infected calf with a bacteremia of >10^7^ *A. marginale* per ml. Following one week of incubation at 37°C to allow blood meal digestion and initial replication, 27 ticks were allowed to attach and transmission feed for seven days on each of the vaccinated or control calves. To confirm that transmission was equal among groups, the ticks were then dissected and the number of *A. marginale* per salivary gland pair determined using *msp5* quantitative PCR as previously described [28]. Challenged calves were monitored daily for *A. marginale* bacteremia by microscopic examination of Giemsa-stained blood smears. Calves that developed anemia, using *a priori* established criteria, were treated with 20 mg/kg long-acting tetracycline and removed from the study. All procedures were approved by the Institutional Animal Care and Use Committee at Washington State University (Approval #2732).

## Results

### AM779 is a sub-dominant antigen in the context of complex immunogens

**Figure 1:**
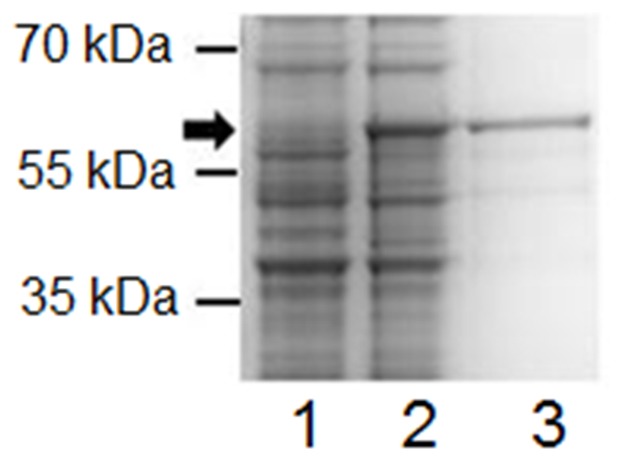
Expression and purification of recombinant AM779. Coomassie stained SDS-PAGE of *E. coli* lysates of uninduced (lane 1) and induced cells (lane 2) expressing AM779, and purified rAM779 (lane 3). The position and size of molecular weight standards is indicated to the left of the image. The arrow designates the position of AM779.

**Figure 2:**
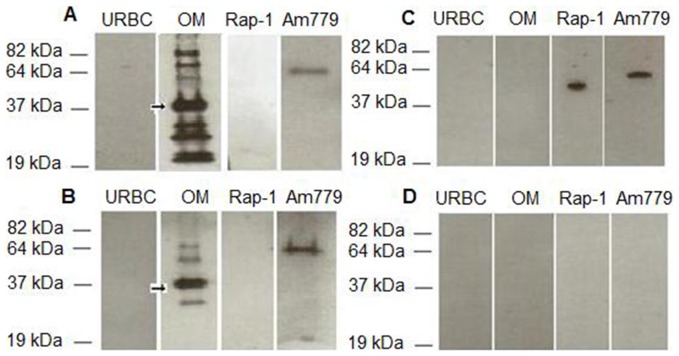
Recognition of AM779 antigen by IgG2 from protected vaccinates. Equal amount of protein (0.4 µg) of the outer membrane immunogen (OM), recombinant full-length AM779, and, as negative antigen controls, uninfected erythrocytes (URBC) and recombinant *Babesia bovis* Rap-1 were electrophoretically separated, immunoblotted and probed for serum IgG2 binding. Sera from *A. marginale* outer membrane immunogen (A) or cross-linked surface protein complex (B) immunized animals were diluted 1∶1000 and tested for binding to AM779. Serum from an adjuvant only immunized animal (D) was used as a negative control. Probing with anti-His antibody was used as a positive control for presence of each recombinant protein domain (C). The position and size of molecular weight standards is indicated to the left of the images and the arrow designates the immunodominant Msp2.

Both isolated native *A. marginale* outer membrane and cross-linked surface protein complexes have been shown to induce protection [7]. These sera were used to determine the IgG2 titer to AM779, using purified recombinant protein (Fig. 1), relative to the complex immunogen. Measurement of IgG2 was specifically chosen as this has been shown to correlate with protective immunity [6]. As shown in Fig. 2A and B, sera from animals immunized with either complex immunogen recognized multiple immunodominant proteins, including Msp2, in antigen preparations of whole bacteria. AM779 is also bound using the same serum dilution (1∶1000) but required a much higher molar ratio of antigen as compared to either complex immunogen or the purified AM779 (Fig. 2A,B). Neither of the two negative control antigens, uninfected erythrocytes nor recombinant *B. bovis* Rap-1 were bound by these sera (2A,B); however Rap-1 was present as shown by the binding of anti-His antibody (2C). There was no reactivity when sera from animals immunized with adjuvant only were used (2D). Using this validated system, IgG2 endpoint titers were determined for AM779 and Msp2 (Table 1). Titers were significantly lower to AM779 than to Msp2 in animals immunized with either complex immunogen (Kruskal Wallis Test for outer membrane vaccinates, p = 0.004; cross-linked surface complex vaccinates, p = 0.007). This difference was not attributable to specific MHC class II haplotypes as the effect was consistent among vaccinates with diverse haplotypes (Table 1). We repeated this analysis with a second set of immunizations, a replicate of the outer membrane vaccinates (n = 5), and observed the same results: there were significantly greater titers to Msp2 (all >20,000) than to AM779 (median, 3,000; mode, 10,000).

**Table 1:** Comparison of titers to AM779 and Msp2 in *Anaplasma marginale* complex immunogen vaccinates.

Animal Number	Vaccine	MHC II haplotypes^a^	IgG2 titer^b^	
			AM779	Msp2
953	OM^c^	16/24	100	>30,000
966	OM	22/24	100	>30,000
975	OM	16/16	100	>30,000
978	OM	24/24	100	>30,000
982	OM	16/8	1000	>30,000
933	CSP^d^	22/24	1000	>30,000
946	CSP	24/24	1000	>30,000
952	CSP	16/24	1000	>30,000
961	CSP	15/24	<100^e^	20,000
972	CSP	16/16	<100^e^	20,000

a Determined by DRβ3 alleles.

b Endpoint titers determined by immunoblotting.

c OM, outer membrane immunized animals.

d CSP, cross-linked surface complex immunized animals.

e Negative at the lowest dilution tested, 1∶100.

### Immunization with AM779 overcomes sub-dominance for T cell but not B cell responses

Using the second set of immunized animals, the AM779-specific T cell responses were measured in both AM779 and outer membrane vaccinates. All outer membrane vaccinates responded, as expected, to the outer membrane antigen; however only 1/5 responded to purified AM779, indicating that AM779 was a very weak immunogen in the context of the membrane complex (Table 2). That this likely reflects relative abundance in the native membrane was supported by the inconsistent response in cells from animals vaccinated with AM779 that were then stimulated with outer membranes (Table 2). In contrast, all AM779 vaccinates responded when stimulated with the autologous antigen. The response was maintained following depletion of CD8^+^ and γδ^+^ T lymphocytes (to 1% and <0.5%, respectively), evidence that the response was CD4^+^ T cell specific (data not shown). All of the animals responded to the positive control clostridial antigen and the cells from adjuvant-only animals were not stimulated by any of the *A. marginale* antigens (Table 2). In contrast to the T cell responses, there was no significant difference in IgG2 titers to AM779 between the outer membrane vaccinates and the AM779 vaccinates either two weeks following the last immunization or immediately pre-challenge. This indicates that for B cell responses, and specifically those leading to class-switching to the relevant opsonizing subclass IgG2 [6],[29], abundance within the complex immunogen is not a primary determinant of sub-dominance.

**Table 2:** Cell mediated responses following immunization with *Anaplasma marginale* complex immunogen or AM779.

Animal Number	Vaccine	MHC II haplotypes^a^	Stimulation Index^b^		
			OM	AM779	*Clostridium*
082	OM	23/22	**2.1**	0.6	**4.2**
100	OM	16/24	**9.3**	**4.0**	**4.8**
108	OM	8/3	**19.1**	1.8	**13.7**
122	OM	24/24	**19.1**	1.2	**13.3**
171	OM	24/24	**2.6** c	1.3	**8.9**
091	AM779	23/24	1.7	**6.3**	**11.3**
113	AM779	16/12	**13**	**21.2**	**127**
117	AM779	8/3	1.7	**6.4**	**34.3**
137	AM779	8/24	**2.4**	**4.3**	**13.5**
149	AM779	24/24	0.9	**3.0**	**24.6**
099	Adjuvant	23/3/27	0.4	0.9	**11.7**
109	Adjuvant	23/27	1.1	1.8	**3.9**
123	Adjuvant	16/3	1.2	0.9	**19.6**
146	Adjuvant	16/8	1.3	1.7	**12.3**
153	Adjuvant	24/24	1.4	1.0	**2.8**

a Determined by DRβ3 alleles.

b Stimulation index (SI) calculated as the mean count per minute (cpm) of triplicate cultures with specific antigen divided by the cpm of triplicate cultures stimulated with the negative control protein Msa-1. Stimulation indices ≥2 were considered significant and are in bold.

c Response was only detected when antigen was used at a final concentration of 3 µg/ml.

### Infectious challenge stimulates an anamnestic response to AM779

Challenge of outer membrane and AM779 vaccinates by feeding *A. marginale* infected ticks represents natural transmission in terms of bacterial structure in the inoculum, the route, and the infectious dose [27]. For animals in both groups of vaccinates, the titers to AM779 increased following challenge (Table 3). The increase was earlier in the AM779 groups in which all animals had significant increases in titer (p = 0.008, one-tailed Mann-Whitney U Test) by one week post-challenge while a similar increase was not observed in the outer membrane vaccinated group until the second week post-challenge.

**Table 3:** Comparison of titers to AM779 and Msp2 in *Anaplasma marginale* complex immunogen vaccinates.

Animal Number	Vaccine	IgG2 titer^a^				
		Msp2	AM779			
		Post-imm.	Post-imm.	Pre-chall.	1 wk Post-chall	2 wk Post-chall.
082	OM^b^	≥20,000	10,000	10,000	10,000	20,000
100	OM	≥20,000	3,000	3,000	3,000	3,000
108	OM	≥20,000	10,000	10,000	10,000	20,000
122	OM	≥20,000	10,000	10,000	10,000	20,000
171	OM	≥20,000	1,000	100	3,000	10,000
091	AM779^c^	-d	3,000	1,000	10,000	10,000
113	AM779	-	10,000	3,000	10,000	10,000
117	AM779	-	10,000	3,000	10,000	10,000
137	AM779	-	3,000	3,000	10,000	10,000
149	AM779	-	1,000	1,000	3,000	3,000
099	Adjuvant	-	-	-	-	3,000
109	Adjuvant	-	-	-	-	10,000
123	Adjuvant	-	-	-	-	3,000
146	Adjuvant	-	-	-	-	3,000
153	Adjuvant	-	-	-	-	3,000

a Endpoint titers determined by immunoblotting at the following timepoints: 1 week following the last immunization (Post-imm.); Immediately pre-challenge (Pre-chall.); 1 week post-challenge (1 wk Post-chall.); 2 weeks post-challenge (2 wk Post-chall.)..

b OM, outer membrane immunized animals.

c AM779, animals immunized with purified recombinant AM779.

d Negative at the lowest dilution tested, 1∶100.

### IgG2 titers to AM779 do not correlate with protection

**Figure 3:**
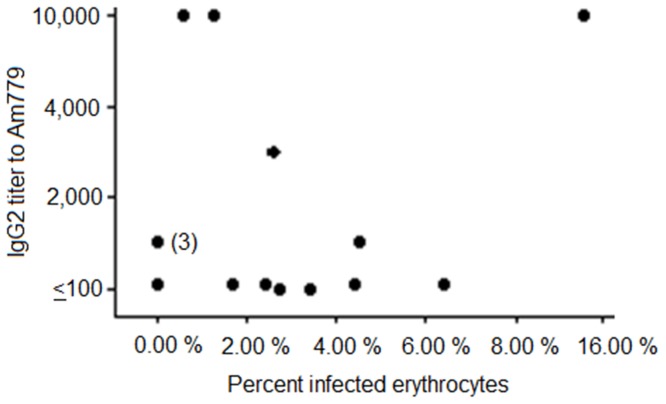
Relationship between IgG2 titer to AM779 and control of bacteremia. Pre-chalIenge IgG2 titers to AM779 were determined for animals immunized with either the outer membrane or the cross-linked surface complex vaccinates. Following challenge, the peak bacteremia, represented as the percentage of *A. marginale* infected erythrocytes, was determined and plotted versus pre-challenge titer. The Pearson Correlation Coefficient was 0.34 and did not reflect a statistically significant relationship. The (3) represents three completely protected vaccinates with the same titer.

Immunization with AM779 did not confer protection against bacteremia: all AM779 vaccinates became infected and had mean peak levels greater than 10^8^ bacteria per ml with infection of >2% of all erythrocytes. The mean peak bacteremia levels were not significantly different from those in the adjuvant control group (p = 0.4; Mann-Whitney Test). To examine the correlation of specific AM779 responses with protection against bacteremia in outer membrane complex and cross-linked surface protein complex, we conducted two analyses. In the first we plotted the bacteremia level, measured as percentage of infected erythrocytes, versus the IgG2 titer to AM779 (Fig. 3). There was no significant correlation (Pearson Correlation Coefficient was 0.34; p = 0.2) between control of bacteremia and the AM779 titer. In the second, we examined whether completely protected vaccinates had higher titers of IgG2 to AM779 as compared to partially protected or unprotected vaccinates. There was no statistical evidence to support this relationship using Kruskal-Wallis analysis (p = 0.26).

## Discussion

Two of the four hypotheses that composed this study were accepted without caveat. The first stated hypothesis, that AM779, a minor outer membrane protein component, is immunologically sub-dominant in the context of the outer membrane and surface protein complex immunogens, was accepted based on both IgG2 antibody and T cell responses. The third stated hypothesis, that AM779 responses are recalled upon presentation of native antigen during actual infection, was also accepted. The second hypothesis, that immunization with recombinant AM779 generates significantly higher responses than when in the membrane context, was accepted for the T cell responses but not for the IgG2 antibody responses. The significantly greater T cell responses when animals were immunized individually with AM779 as compared to animals immunized with the outer membranes were observed independent of the MHC class II haplotypes of vaccinates. This is consistent with the presence of multiple immunogenic epitopes in AM779 that can be presented via the diverse MHC class II molecules represented within the immunized population. These results suggest that for AM779 relative abundance in the immunogen may be deterministic for T cell responses rather than intrinsic epitope structure being responsible for sub-dominance relative to major components such as Msp2 and Msp3.

The anamnestic response upon challenge with *A. marginale* indicates that while of low abundance in the bacterial outer membrane, there is sufficient antigen to stimulate memory B lymphocytes, cells which due to high affinity immunoglobulin receptors induced by prior immunization have a much lower antigen requirement for reactivation [30]. Interestingly, the AM779 specific recall response was detected earlier in the AM779 vaccinates than in the outer membrane vaccinates, suggesting that there may have been differences in the vaccine induced priming between the two groups. This is consistent with the more uniform AM779 specific T cell response in the AM779 vaccinates which may provide more robust help upon re-exposure to the antigen. In addition, the anamnestic response to infectious challenge in the animals immunized with AM779 supports that the recombinant immunogen faithfully represents native epitope structure. Importantly, the challenge was via feeding of infected ticks and thus both the infectious dose and bacterial structure were representative of natural transmission.

AM779 responses, whether induced by immunization with recombinant AM779, outer membranes, or surface complexes, did not associate with protective immunity. Thus, we reject the fourth tested hypothesis. While lack of protection in experimental vaccine trials is always a disappointment, this reporting is as important as for successful trials. The number of variables involved in immunization (adjuvant, antigen dose, route of delivery, number of boosters, vaccine interval, etc.) make it difficult to conclude that a specific antigen, in this case AM779, should no longer be considered a viable vaccine candidate. Nonetheless, we can conclude that even with AM779 specific titers that significantly exceed those induced in outer membrane or surface complex immunized animals, AM779 by itself is not protective.

Whether a single sub-dominant antigen can protect against infection with *A. marginale* or related pathogens is unresolved. AM779 was identified as being a component of three protective immunogens: outer membranes, surface complexes, and the live *A. marginale* ss. *centrale* vaccine strain [4]. However all three of these protective immunogens are themselves complex. The outer membrane is composed of 21 identified proteins that induce IgG2 in vaccinates while the surface complexes contain 11 proteins [4],[7],[19]. The live vaccine strain, of course, has the full complement of outer membrane proteins, estimated from combined bioinformatics and proteomic analyses to exceed 60 [18],[19],[22],[31]. Consequently, in conceptualizing vaccines for *A. marginale* and related pathogens, it may be helpful to borrow definitions from molecular pathogenesis. In this view, sub-dominant antigens such as AM779 may be “required” but “not sufficient” to induce protective immunity. Inducing uniform protection among vaccinates using complex immunogens such as the outer membrane and surface complexes may require augmentation with specific individual membrane proteins in order to overcome the sub-dominance attributed to their low abundance or intrinsic lack of epitope density. Importantly, immunization with AM779 supports that once priming is achieved by the increased antigen dose, recall upon infectious challenge is achieved. This supports continued investigation into the role of sub-dominant antigens, individually and collectively, in vaccine development for *A. marginale* and related bacterial pathogens.
